# Who Leads and Who Follows? The Pathways to Joint Attention During Free‐Flowing Interactions Change Over Developmental Time

**DOI:** 10.1111/cdev.14229

**Published:** 2025-02-18

**Authors:** M. Perapoch Amadó, E. A. M. Phillips, G. Esposito, E. Greenwood, J. Ives, P. Labendzki, K. Lancaster, T. J. Northrop, N. K. Viswanathan, M. Gök, M. J. Peñaherrera, E. J. H. Jones, S. V. Wass

**Affiliations:** ^1^ University of East London London UK; ^2^ Birkbeck University of London London UK

**Keywords:** joint attention, mother‐infant dynamics, naturalistic research

## Abstract

Joint attention (JA) has been found to correlate with many developmental outcomes. However, little is known about how naturalistic JA is established and develops during early infancy. In this study, free‐flowing tabletop toy play between infants at 5 and 15 months and their mothers (*N* = 48 dyads; 65% white) was observed to (1) examine changes in JA, (2) investigate whether infants become better leaders or followers of JA, and (3) explore the role of intentionally mediated forms of communication. JA episodes increased in frequency and duration, and initiations of JA became more evenly distributed between members of the dyad. Older infants became better at leading as well as following their mothers' attention behaviors and more frequently directed their attention towards their partner, though this had minimal impact on the organization of episodes of JA.

From about 3 to 6 months old, infants begin to be able to coordinate their attention with a social partner in connection to a separate object or event (Butterworth 2001; Carpenter et al. 1998; Corkum and Moore 1998; Moore et al. 2014; Mundy and Newell 2007; Mundy and Sigman 2015). In the literature, this phenomenon is widely known as joint attention (JA). Overwhelming evidence correlates the ability to coordinate visual attention with others to an object or event with many developmental outcomes, including language learning (Mundy and Newell 2007; Yu and Smith 2013), social learning (Mundy and Newell 2007), and other broader cognitive skills (Bornstein 1985; Schroer and Yu 2022). But how do infants become capable of coordinating their attention with others? Surprisingly, despite universal agreement about the importance of JA as the main source of learning opportunities in infancy, there is still little agreement on exactly how the *jointness* of JA is achieved and what its theoretical underpinnings are.

Some authors understand JA as “looking where someone else is looking” (Butterworth 2001). Others, instead, emphasize the importance of internal models about the mental state of others. They highlight the importance of shared intentionality (Carpenter et al. 1998; Tomasello and Carpenter 2006) and argue that to be in JA, both individuals must not only be experiencing the same thing at the same time, but “they must know together that they are attending to the same thing” (i.e., they must have common knowledge; Tomasello and Carpenter 2006). From this perspective, JA involves using communication cues to guide and share attention with the partner.

Current understanding shows that episodes of JA become more frequent as development progresses (e.g., Aureli et al. 2022; Bakeman and Adamson 1984), but the mechanisms underlying these changes are still unclear. Response to and initiation of JA have been reported as the main components. Responding to JA (RJA) refers to infants' ability to follow the direction of the gaze and gestures of others to share a common point of reference. Alternatively, initiating JA (IJA) involves infants' use of gestures and eye contact to direct others' attention to objects, to events, and to themselves (Mundy et al. 2007; Mundy and Newell 2007). Research in the field has often examined how these skills develop through screen‐based tasks involving eye‐tracking or employing standardized tests such as the Early Social‐Communication Scales (ESCS; Mundy et al. 2003). Generally, the development of RJA is thought to begin early (Scaife and Bruner 1974) and is characterized by significant improvements in accuracy during the initial year of life (Jones et al. 2014; Morales et al. 2000; Mundy 2018). For example, infants younger than 12 months can follow an adult's head turn correctly but are unable to accurately locate the target that the adult focuses on when multiple targets are present (Butterworth and Jarrett 1991) or when the object is out of sight (Delgado et al. 2002; Moll and Tomasello 2004). Similarly, infants from around 10 to 11 months, but not younger, followed a head turn when the person's eyes were open but not when they were closed (Brooks and Meltzoff 2005). IJA, instead, is believed to start developing later during the second half of the first year of life (Billeci et al. 2016; Mundy 2018). From around 8 to 9 months, infants start to develop the ability to use pointing and gaze to initiate episodes of JA, alternating their direction of gaze between a person and an object to share engagement (Carpenter et al. 1998; Mundy 2018).

Two competing theories focus on infants being either passive or active contributors to JA in social interactions. On the one hand, the theory of natural pedagogy suggests that human communication is specifically adapted to allow the transmission of generic knowledge between individuals and argues that human infants are prepared to be at the receptive side of such transmission (Csibra and Gergely 2009, 2011). The most obvious ostensive signal in human communication is direct gaze towards the addressee (Csibra and Gergely 2009). In line with this, studies have found that even newborns exhibit a preference for faces with direct gaze as opposed to averted gaze (Farroni et al. 2006). As discussed before, while the exact age remains a topic of discussion, it is generally accepted that the ability to follow a social partner's gaze develops significantly during the first year of life (e.g., Brooks and Meltzoff 2005; Butterworth and Jarrett 1991; Farroni et al. 2004; Flom and Johnson 2011; Gredeback et al. 2008). Consequently, one possibility is that infants' ability to detect and respond to ostensive signaling from adults improves with time, thereby improving their responsiveness to JA.

Alternatively, active learning theories view infants as proactive seekers of information. For example, infants, through social referencing, babbling, and pointing, selectively seek, elicit, and modulate the information they receive from informative social partners (e.g., Begus and Southgate 2012, 2018; Gottlieb and Oudeyer 2018). Guided by curiosity, infants use ostensive behaviors to actively direct their partners' attention to receive new information about their environment. For example, Liszkowski et al. (2004) showed that 12‐month‐olds point more when the adults actively share their attention and interest with them than when they do not (Liszkowski et al. 2004). In recent studies, it has been shown that 12‐month‐olds display increased pointing behaviors when provided with feedback that provides new information about an object, as opposed to situations where the experimenter merely shares attention and interest with the infant (Kovács et al. 2014). Additionally, 16‐month‐olds exhibit similar behaviors, pointing more toward adults perceived as competent in labeling objects compared to those perceived as ignorant (Begus and Southgate 2012). Together, these results fit well with the idea that infants expect to learn something from the response they receive to their ostensive behaviors (Southgate et al. 2007). Importantly, these ostensive behaviors used by infants to initiate JA may also be used for less instrumental but more social purposes (Bates et al. 1976 in Mundy and Sigman 2015; Mundy 1995). Either way, the adult dynamically adapts to the child but not *vice versa* (Begus and Southgate 2012, 2018; Wu and Gros‐Louis 2015). From these perspectives, as infants get better at signaling intention, their ability to initiate JA would also improve.

Most of this research, however, has used structured tasks (Northrup and Iverson 2020). While useful, these contexts are quite distinct from unstructured social interactions in which JA “is embedded in a stream of free‐flowing activity in which parents both react to and attempt to control toddlers' behaviours and toddlers react to, direct, and sometimes ignore parents as they pursue their own goals” (Northrup and Iverson 2020; Yu and Smith 2017). The repetitive nature of the tasks might prompt infants to exhibit behaviors that they would otherwise not typically display in naturalistic settings, where partner behaviors unfold within a continuum of multimodal and complex dynamics. This could potentially limit the generalizability of the findings (Tang et al. 2023). Furthermore, structured tasks such as the Early Social‐Communication Scales (Mundy et al. 2003) require adult experimenters to perform tasks in a pre‐specified order, which obviates the possibility that developmental changes may be observed not just due to maturational changes in the child but also due to changes in the partner too (e.g., that parents become more skilled over time in directing a child's attention). It remains possible, then, that other characteristics from the adult partners such as increased perceptivity (i.e., ability to understand their infants) from the mothers to older infants (Thorson et al. 2018) or reduced leadership (which would allow the infant to take more of a leading role in initiating JA; Evans and Porter 2009; Kochanska and Aksan 2004) might also contribute to establishing infant‐led JA.

As a result of these limitations, significant efforts have been made to study the micro‐dynamics of infant attention while they engage in more naturalistic, free‐flowing interactions with their caregiver (e.g., Abney et al. 2020; Phillips et al. 2023; Yu and Smith 2013). This has allowed researchers to both overcome important limitations from previous research, such as studying infants in isolation or within highly structured experimental paradigms, and significantly improve our understanding of the second‐by‐second dynamics and influences between infant–mother dyads (Phillips 2023; Wass et al. 2018; Yu and Smith 2013).

In these more naturalistic contexts, socially coordinated shifts in attention are resolved in fractions of a second (Phillips et al. 2023; Yu and Smith 2013). For example, several studies have now found that infants spend only a small proportion of time looking to their parent's face (Franchak et al. 2011; Phillips et al. 2023; Yu and Smith 2013). Phillips et al. (2023) compared infant‐led looks to JA and non‐JA in 10‐month‐olds and found no differences in infants' use of behaviorally ostensive cues in the 5 s window before the initiation (i.e., looks to parents' face and vocalizations prior to leading a look), indicating that infants do not appear to direct the focus of their attention deliberately and actively when they guide the attention of their partners. Similarly, Yu and Smith (2017) studied JA in 12‐ and 18‐month‐old infants and found that when toddlers followed their parents' attention, they rarely did so by gaze following (< 15%) but instead typically followed their parents' hands to the object (Yu and Smith 2017): a spatially and attentionally simpler, and thus, faster, pathway to JA, compared to gaze following. Together, these studies (and others, e.g., Yu and Smith 2013) suggest that JA is a self‐organizing outcome built upon the multimodal coupling of partners' individual sensory‐motor behaviors (Yu and Smith 2013). In other words, rather than controlled, intentionally mediated ostensive signaling, it is the fast‐acting, sensory‐motor coordination of both partners that largely drives and maintains episodes of JA.

Despite these insights, our understanding of how different leader‐follower processes function within the dyad and evolve over early developmental time to change JA remains limited. Similarly, no study has yet explored how intentionally mediated ostensive signals, such as “looks to the parent”, change over time. Therefore, our study aims to: (1) examine developmental changes in quasi‐naturalistic JA, (2) explore whether these are driven by infants becoming better initiators of new attention episodes (i.e., better leaders, IJA) or by their improved coordination with play partners (i.e., better followers, RJA), and (3) investigate whether these changes are driven by developments in intentionally mediated forms of communication (i.e., infants engaging in ostensive signals such as looks to partner) or not.

To do this, we manually coded the gaze of mothers and their 5‐ and 15‐month‐old infants as they jointly played with toys (see Section 1.2 for more details). These ages were chosen because this is an age range where structured tasks have shown dramatic changes in both RJA and IJA (Mundy and Newell 2007). During this time, infants acquire numerous new skills, encompassing cognitive, communicative, and motor abilities (Feldman 2007; Yu and Smith 2013). Additionally, according to Tomasello and colleagues (e.g., Tomasello 2001) it is also the time in which the ability to share intentionally mediated JA starts to emerge. By studying these ages, we aim to understand how the mechanisms that drive JA change over this unexplored time period. By tracking the momentary visual fixations of each participant, we determined the frequency (count) and average duration of different types of attentional looks (Analysis 1). This also allowed us to determine how infants and their mothers enter moments of JA (i.e., by following their partner or leading them into JA) and how these dynamics changed over time (Analysis 2). We also explored the sensitivity of one partner to changes in behaviors generated by the other partner (Analysis 3) and examined the use, contributions, and influences of ostensive signals (i.e., looks to the partner) in establishing and organizing episodes of JA (Analysis 4).

Hypothesis 1 is that JA is increasingly infant‐driven. We reasoned that, if this was true, we would observe an increase over time in infant leader looks to JA (i.e., increase in IJA) (Analysis 2). We also reasoned that mother's looking behavior would be progressively more reactive to infants' looking behavior over time (Analysis 3).

An alternative hypothesis, Hypothesis 2, is that developmental changes in JA are driven by infants becoming better at responding to their partner's behavior. We reasoned that, if this was true, we would observe an increase in infant follower looks to JA (i.e., increase in RJA) (Analysis 2). We also reasoned that infant's looking behavior would be progressively more reactive to mothers' looking behavior over time (Analysis 3).

In both scenarios, it was anticipated that improved leading or following abilities would lead to a greater chance of infants coordinating their attention with their mothers, resulting in an increase in JA episodes over developmental time.

Finally, we asked whether these changes would be facilitated by the dyad increasingly engaging in ostensive signals such as looks to partner or not (Analysis 1 and 4). Based on previous research, we hypothesized that older infants would indeed show more intention to involve others (Hypothesis 3; Carpenter et al. 1998; Mundy et al. 2007; Tomasello and Carpenter 2006) and reasoned that this strategy would enable them to ensure that partners are indeed directing their attention to the same object and verify both shared intention and attention to the same object. Accordingly, we predicted that intentional ostensive signals such as infant looks to partner would increase with time (Analysis 4.1) and that looks to partner *before* leader looks would contribute to infants becoming better at leading and following their partner into JA more efficiently (Analysis 4.2).

## 1 Materials and Methods

### 1.1 Participants

Participants were typically developing infants and their mothers. The catchment area for this study was East London, including boroughs such as Tower Hamlets, Hackney, and Newham. Further demographic details on the sample are given in Table S1.

Participants were recruited postnatally through advertisements at local baby groups, local preschools/nurseries, community centers, and targeted social media campaigns aimed at all mothers in the area. We also operated a word‐of‐mouth approach. Ethical approval was obtained from the University of East London ethics committee (application ID: ETH2021‐0076). Data collection for the current study took place between August 2021 and July 2023.

Initial exclusion criteria include complex medical conditions, known developmental delays, prematurity, uncorrected vision difficulties, and mothers below 18 years of age. Further exclusion criteria as well as final numbers of data included in each of the analyses for both samples are summarized in Table S2. The final samples included 24 5‐month‐old infants (11 females) and 24 15‐month‐old infants (11 infant females) and their mothers. Data were analyzed in a cross‐sectional manner. Average age for infants was 5.3 months (std = 0.55) and 15.77 months (std = 0.87), respectively. Average age for mothers was 35.24 years (std = 4.29, *N* = 23) at 5 months and 36.93 years (std = 4, *N* = 24) at 15 months. This is the first time that any of these data have been analyzed and reported.

### 1.2 Experimental Design

**FIGURE 1:**
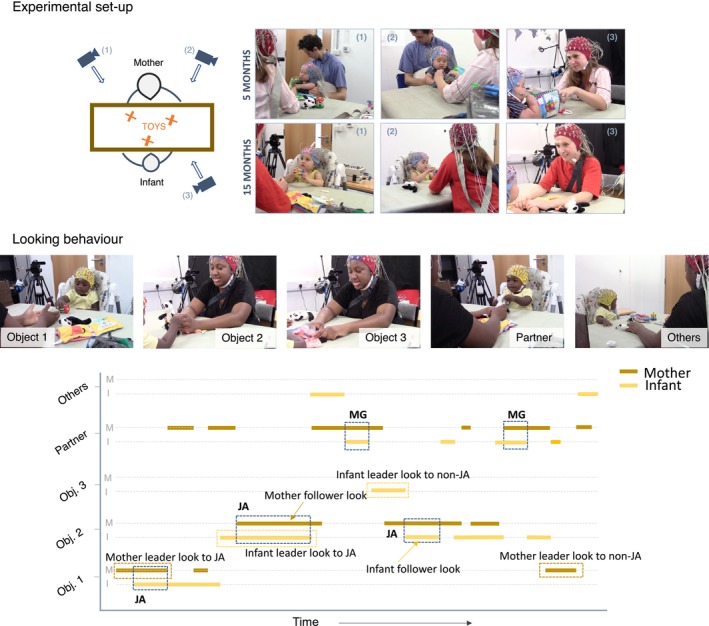
Experimental set up and an example of looking behavior. Top figure shows the experimental set up for the joint play condition. Two cameras pointed at the infant (view in photos 1 and 2) and one camera pointed at the mother (view in photo number 3). Looking behavior was coded manually for object and partner looks from both the mother and the infant. Photos in the middle row show the different type of looks (i.e., looks to object 1–3, looks to partner and ‘others’. Notice that the latter category—‘others’—included inattention and uncodable moments). Bottom figure shows an example of the time series data and how the main look categories (i.e., leader looks to non‐JA, leader looks to JA and follower looks) are defined. JA = Joint attention, MG = Mutual gaze.

Mothers and infants were seated facing each other on opposite sides of a table. Infants were seated either in a highchair or on a researcher's lap, within easy reach of the toys (see Figure 1). At the beginning of the joint play session, a researcher placed the toys on the table and asked the mothers to “play with their infants just as they would at home”. During the play session, researchers stayed behind a divider out of view of both the mother and the infant. The same three toys were used for each age group (see Figure S1). The average duration of the joint play interactions was 4.94 min (std = 1.36) at 5 months and 6.47 min (std = 1.44) at 15 months. Average duration differed significantly between 5‐ and 15‐months (t(46) = −3.778; *p* < 0.001). Given that the analyses are conducted relative to the duration of each interaction (e.g., look counts per minute) or on specific events (e.g., the average durations of looks at objects), variations in interaction durations should not be an issue.

The interactions were filmed using three Canon LEGRIA HF R806 camcorders recording at 50 frames per second (fps). Two cameras were placed in front of the infant, one on each side of the mother, and another was placed in front of the mother, just behind the right side of the infant. All cameras were positioned so that the infant's and the mother's gaze, as well as the three toys placed on the table, were always visible (see Figure 1). Of note, brain activity was also recorded from both the infants and their mothers, at both ages, using a 64‐channel BioSemi gel‐based ActiveTwo system. However, these data are not included in the current manuscript.

### 1.3 Data Processing

#### 1.3.1 Synchronization of the Video Data

The cameras pointing at the participants were synchronized via radio frequency (RF) receiver LED boxes attached to each camera. The RF boxes received trigger signals from a single source (computer running Matlab) at the beginning and end of the play session, and concurrently emitted light impulses visible from each camera and an audible beep. The synchronization of the video coding for maternal and infant behavior was conducted offline by aligning the times of the LED lights of the three cameras and checking that the durations matched.

#### 1.3.2 Gaze Behavior Coding and Processing

The looking behavior of the infants and their mothers was manually coded offline on a frame‐by‐frame basis, at 50fps (see Figure S2 for an example of raw data). The start of a look was the first frame in which the gaze was static after moving to a new location. The following categories of gaze were coded: looks to objects (focusing on one of the three objects), looks to partner (looking at their partner), inattentive (not looking to any of the objects nor the partner) and uncodable (see Figure 1). Uncodable moments included periods where: (1) their gaze was blocked or obscured by an object and/or their own hands, (2) their eyes were outside the camera frame, and/or (3) a researcher was within the camera frame. Of note, inattentive looks and uncodable moments were subsequently grouped together into a category labeled as ‘others’.

To assess inter‐rater reliability, ~15% of the data (13 datasets) were double coded by a second coder and Cohen's kappa was calculated. There was substantial agreement (*κ* = 0.636, std. = 0.143; Landis and Koch 1977). Looking behavior data were then processed such that any look preceding and following an “uncodable” period was excluded from further analyses. Similarly, both the first and the last look of every interaction were also excluded from further analyses.

##### 1.3.2.1 Processing Leader and Follower Looks

Similar to other studies (Phillips et al. 2023; Yu and Smith 2013), we interpolated through infant and mother looks to their partner before examining leader‐follower dynamics (Analysis 2 and 4). This is because, during periods of JA towards an object, mothers and, to a lesser extent, infants alternated their attention frequently between the object and their partner. Without interpolation, each subsequent look back to the object would be classified as a separate follower look to the object. This procedure, therefore, allowed us to accurately identify moments where one partner was leading or following their partner's attention. Therefore, unless specified otherwise, any analysis involving leader/follower looks will be done using interpolated data.

More specifically, interpolation involved identifying moments where the mother or infant directed their gaze towards a specific object, subsequently shifted their attention to their partner for less than 2 s, and then reverted to focusing on the initial object. By interpolating through that partner look, the partner look became an extension of the preceding and following object look. Given that previous studies have used a range of different minimum thresholds for interpolation (Phillips et al. 2023; Yu and Smith 2017), we also conducted a sensitivity analysis in which we repeated our analyses using the same threshold of 300 ms used by Yu and Smith (2017, see Figure S3). The results observed were the same as those reported in the main text.

After interpolation, the first and last frame of all looks to the object were extracted and categorized into one of our look categories: leader or follower looks. Leader looks were defined when one of the partners shifted their gaze towards an object that the partner was not already looking at. These looks were further divided into two categories: leader looks that led to JA (these were the leader looks that were subsequently joined by the partner) and leader looks that did not lead to JA (these were leader looks that were not joined by the partner). Follower looks, on the other hand, were defined as those looks that followed the partners' attention (see Figure 1). JA was defined as the periods of time when both partners were looking at the same object at the same time (refer to Table S3 for a detailed description of each look and attention categories). Similar to other studies (e.g., Phillips et al. 2023; Yu and Smith 2013), there was no threshold for how long partners needed to be looking at the same object to count as JA.

#### 1.3.3 Calculation of significance: Cluster‐based permutation (CBP) test

To estimate the significance of the time‐series relationships in analyses 3 and 4.2, we chose a cluster‐based test statistic and used the so‐called Monte Carlo method to calculate significance. To do so, we used a function from FieldTrip (Maris and Oostenveld 2007) called “ft_timelockstatistics”. This nonparametric framework allowed us to both control for the multiple comparison problem that arises from the fact that the effect of interest is evaluated many times (e.g., infant attention around mother looks to the partner) and to reduce the potential for false negative effects (Meyer et al. 2021).

## 2 Results

In this study, we investigated developmental changes in quasi‐naturalistic JA between infants and their mothers. First, we asked whether infants, as their attention control improves over time, would become more adept at leading (Hypothesis 1) or following (Hypothesis 2) their partner into JA. Second, we asked whether these changes would be driven by developments in intentionally mediated forms of communication (i.e., infants engaging more in ostensive signals such as looks to partner) (Hypothesis 3). To test this, we first explored the frequency (count) and average duration of different types of looks (i.e., looks to objects, looks to partner and inattention) (Analysis 1). Next, in Analysis 2, we determined how infants and their mothers enter moments of JA (i.e., by following their partner or leading them into JA) and how these dynamics changed over time. In Analysis 3, we explored the sensitivity of one partner to changes in behaviors generated by the other partner. Finally, in Analysis 4, we examined the use, contributions, and influences of ostensive signals (i.e., looks to the partner) in establishing and organizing episodes of JA.

### 2.1 Analysis 1. Descriptive Analyses on Gaze Behavior

**FIGURE 2:**
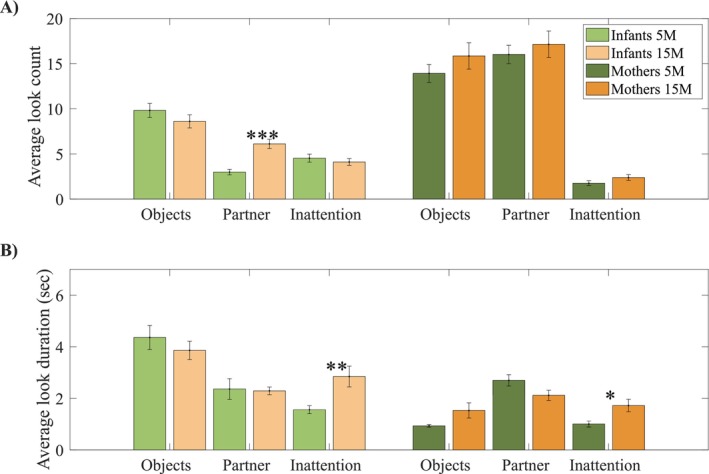
Descriptive analyses on looking behaviour. Figure showing average number of looks per minute (A) and duration (in seconds) (B). Asterisks indicate significance (*p < 0.05, **p < 0.01, ***p < 0.001).

In Analysis 1, our main aim was to explore between‐ and within‐age group differences in how many times per minute infants and mothers engaged in attention to play objects, partner, or inattention (Figure 2A) and for how long these attentional episodes lasted on average (Figure 2B).

To compute these analyses, we used non‐interpolated data. To calculate the average frequency (count; Figure 2A) of attention reorientations, we calculated the occurrence of each of these types of looks and divided it by the length of the session.

During early infancy, infants shifted their attention frequently between play objects (*M* = 9.82, std. = 3.82; object looks per minute) and rarely looked at their partners (*M* = 2.99, std. = 1.52; partner looks per minute; see Figure 2A and Figure S1). As infants grew, these dynamics changed. Older infants looked significantly more frequently at their partner (*M* = 6.11, std. = 2.51; partner looks per minute) (*t*(46) = −5.204, *p* < 0.001) compared to younger infants (Figure 2A and S2), and less frequently at the play objects (*M* = 8.61, std. = 3.56; object looks per minute), though the difference in object‐looking was not statistically significant (*t*(46) = 1.14, *p* = 0.259). Periods of inattention became less common with age (*M*_5M_ = 4.54, std. = 2.17; and *M*_15M_ = 4.11, std. = 1.87; inattention looks per minute), though this difference was not statistically significant. However, when inattention occurred, it lasted significantly longer (*M*_5M_ = 1.57 s, std. = 0.76; and *M*_15M_ = 2.85 s, std. = 1.97) (*t*(46) = −2.968, *p* < 0.01; Figure 2B). The gaze behavior of mothers, instead, remained stable between the two time points with some variation in the duration of inattention, which increased from 5 months (*M* = 1.01 s, std. = 0.55) to 15 months (*M* = 1.72 s, std. = 1.19) (*t*(44) = −2.568, *p* = 0.013). Differences in attention duration to objects and to partners were marginally not significant (*t*_objects_dur_(45) = −1.95, *p*_objects_dur_ = 0.057; *t*_partner_dur_(45) = 1.97, *p*_partner_dur_ = 0.055).

### 2.2 Analysis 2. Dynamics Between Leader and Follower Looks

Here we investigated the dynamics of three different types of looks (see Table S3): leader looks to objects that were not followed by the partner (leader looks to non‐JA), leader looks to objects that were followed by the partner and led to JA (leader looks to JA), and object looks that followed their partner's attention and thus also led to JA (follower looks). If infants become better leaders over developmental time (Hypothesis 1), we predicted an increase in leader looks to JA. Conversely, if they become better at following their partners into JA (Hypothesis 2), we predicted an increase in follower looks.

**FIGURE 3:**
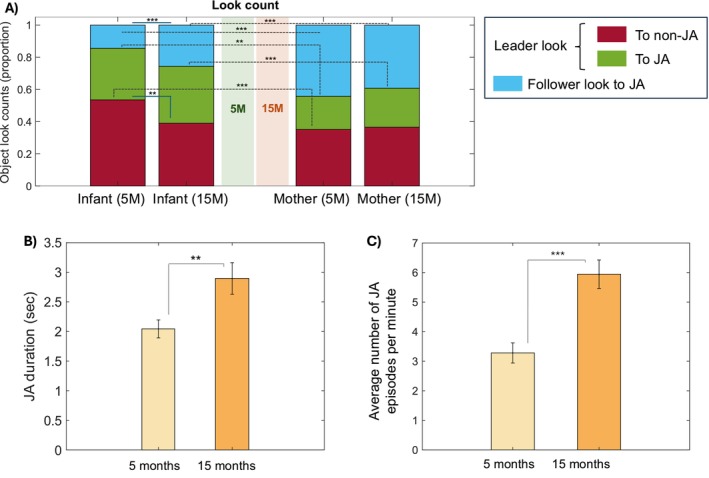
Dynamics between leader and follower looks. (A) Figure showing the proportion of leader looks to non‐JA (in red), leader looks to JA (in green) and follower looks to JA (in blue). Infant (left) and mother (right). (B) Shows the average duration of JA and (C) shows the average number of JA episodes per minute. Asterisks indicate significance (*p < 0.05, **p < 0.01, ***p < 0.001). Analyses are done using interpolated data.

To test this, we first calculated the proportion of these looks and compared them across the dyad members and the different ages (Figure 3A). We observed that, at 5 months, when infants do lead an attention shift, this generally does not lead to JA with their partners (Figure 3A). The same was true for infants at 15 months. However, during later infancy, there was a significant increase in infant leader looks that did lead to JA relative to infant leader looks that did not lead to JA (i.e., proportion of infant leader looks to JA at 5 vs. 15 m increased: *t*(44) = −2.199, *p* = 0.033). Older infants also performed significantly fewer leader looks to non‐JA (*t*(44) = 3.494, *p* = 0.001). Refer to Figure S4 to see the average number of looks per minute. At both ages, most of the JA moments were driven by the mother responding to the infant's initiations of attention (Figure 3A). However, with time, infants became more able to also follow their partners (i.e., proportion of infant follower looks at 5 vs. 15 months increased: *t*(44) = −4.899, *p* < 0.001; Figure 3A) and, as a result, the initiations of JA became more equally distributed within the members of the dyad (i.e., proportion of mother leader looks at 5 vs. 15 m increased: *t*(44) = 2.221, *p* = 0.031). Interestingly, the distribution of these different type of looks in the mothers' data remained consistent across both time points.

Next, we looked at the duration and frequency (i.e., average count per minute) of JA episodes. The results showed significant increases in both the duration (*t*(44) = −2.781, *p* = 0.007; Figure 3B) and the average frequency of JA episodes per minute (*t*(44) = −4.517, *p* < 0.001; Figure 3C) over developmental time.

Overall, we observe that at 15 months, infants' initiations are more likely to lead to JA with their partner compared to 5 months, and they engage in more follower looks (Figure 3A). Consequently, JA episodes become longer and more frequent (Figure 3B,C). We repeated this last analysis using a different time threshold for interpolation (see Section 1.3.2.1); results stayed the same (see Figure S3).

### 2.3 Analysis 3. Responsivity to Changes in Partner Gaze Behavior

Next, we looked at responsivity to changes in the partner looking behavior. More specifically, we looked at how an individual responded to different types of looks initiated by their partner. In Analysis 3.1, we examined how they responded to looks towards the partner, while in Analysis 3.2, we examined how they responded to looks towards the objects. Studying behaviors at the event level is important as it allows us to gain a better understanding of proximate mechanisms and influences (Schroer and Yu 2022). If infants become better leaders over developmental time (Hypothesis 1), we anticipate that changes in the infants' looking behavior would have a greater influence on mother's attention as they lead them more into JA. Conversely, if they become better at following their partners into JA (Hypothesis 2), we anticipate that changes in the mothers' looking behavior would have greater influences on infant attention as they follow them more into JA.

#### 2.3.1 Analysis 3.1. Responsivity to Partner Looks

Here, we studied whether changes in the mothers' looking behavior towards their infants would have a greater influence on the infant's attention over time, and similarly whether changes in the infant's looking behavior towards their mothers would have a greater influence on the mother's attention over time. To start with, we first selected all mother looks to the partner (i.e., infant) and took the onset of each of these looks. Then, we took the infant data and time locked it to each of the mothers' look‐to‐partner onsets. Third, we selected a 20‐s segment of infant data before and after each onset. To study how infant *attention to objects* changed around moments when the mothers looked at their infants, we assigned ‘1’ to each frame within the 20‐s segment where the infant was looking at objects, and ‘0’ to each frame where the infant was not (e.g., partner, inattention, etc.). Next, we calculated the proportion of looking at the objects by summing all the 1 s across events (i.e., maternal looks to the infants) and subjects and dividing it by the total number of events. To study how *attention to* *partner* changed around moments when the mothers looked at the infant, we assigned ‘1’ to each frame where the infant was looking at the partner, and ‘0’ to any other infant look.

Then, we looked at the inverse relationship by first selecting all infant looks to the partner (i.e., mother) and taking the onset of each of these looks. Then, we took the mother data and time‐locked it to each of the infant's look‐to‐partner onsets. In this way, we studied maternal responsivity to infant looks to the partner.

To compare whether the observed responses were significantly higher than chance, we generated control data. To do so, we followed the same steps as above with the observed (real) data with one distinction: instead of using the original events (e.g., times when mothers looked at the infants), we selected 200 random times throughout each interaction and used these as events to create the control data. To compare the observed responses with the control responses, we employed a CBP test (see Section 1.3.3). Similarly, to compare the observed responses between the two time points (5 vs. 15 months) we also employed a CBP test. Prior to this, we applied a baseline correction to facilitate the comparison across the two time points. To do this, we averaged the data from the first 10 s of each 40‐s segment and subtracted it from the rest of the 40‐s segment.

**FIGURE 4:**
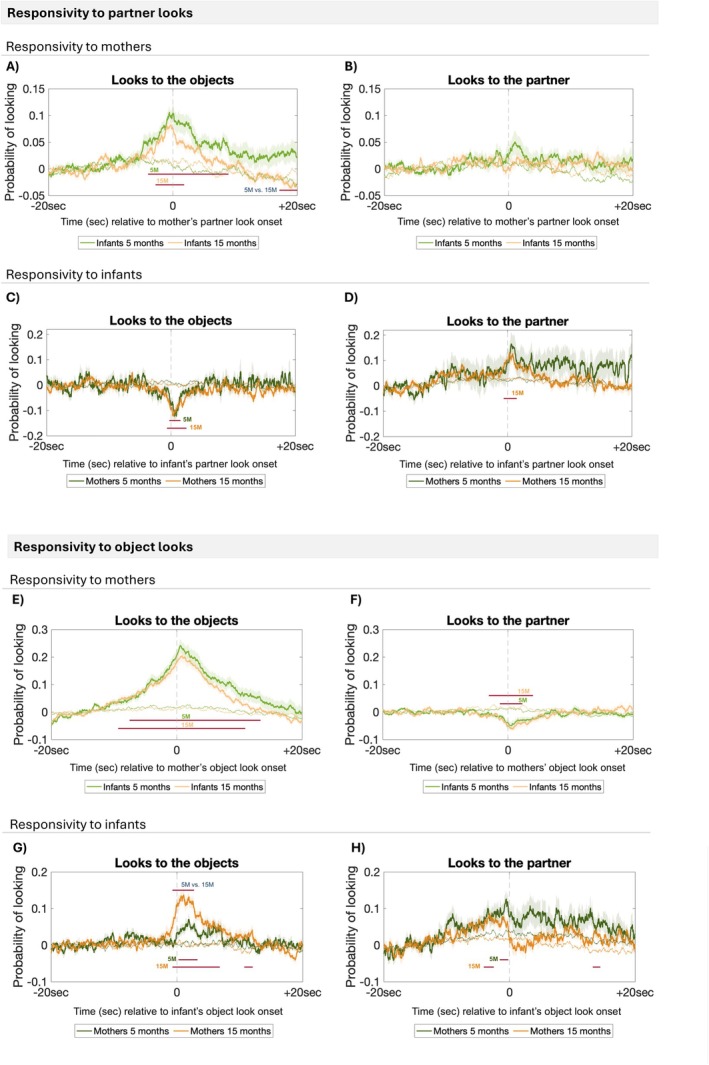
Figure showing responsivity to changes in partner gaze behavior. (A–D) responsivity to partner looks. Probability of infants (A) and mothers (C) to look at the object when their partner looks at them. Probability of infants (B) and mothers (D) to look at the partner when their partner looks at them. (E–H) responsivity to object looks. Probability of infants (E) and mothers (G) to look at the same object as their partners when their partner looks at the object. Probability of infants (F) and mothers (H) to look at the partner when their partner looks at the objects. Green thick lines indicate infants (light green) or mothers (dark green) data at 5 months. Orange thick lines, instead, indicate infants (light orange) or mothers (dark orange) data at 15 months. Thin lines are control data at 5 months (green) or 15 months (orange). Red thick lines indicate significance from the CBP test (*p* < 0.025, two‐sided). Analyses are done using non‐interpolated data.

We found no age effects in the way infants or mothers responded to partner looks. However, the results showed some interesting infant/mother asymmetries. For infants at both ages, the probability of looking at the object was significantly higher around (and after) moments when their mothers were looking at the infant (CBP test indicated that there was a significant difference between observed and control data, *p*_5M_ < 0.001, *p*_15M_ < 0.001; Figure 4A). As a likely consequence, we observed that infants, even at 15 months old, did not respond by looking back at their mothers when their mothers looked at them (the CBP test revealed no significant clusters; Figure 4B). For mothers, instead, the probability of looking at the object decreased around moments when the infant looked at them (CBP test, *p*_5M_ < 0.001, *p*_15M_ < 0.001; Figure 4C). We also observed that mothers with older, but not younger, infants responded when infants looked at them by looking back at their infants (CBP test, *p*_15M_ = 0.002; Figure 4D).

#### 2.3.2 Analysis 3.2. Responsivity to Object Looks

Here, we studied whether changes in the mothers' looking behavior towards objects would have a greater influence on the infant's attention over time; and, similarly, whether changes in the infant's looking behavior towards objects would have a greater influence on the mother's attention over time. We first looked at how the probability of mothers and infants looking at the *same* object changed in response to their partners' attentional shifts towards the objects. To do so, we took the onsets of all the mother looks to Object 1. Second, we time‐locked these maternal look onsets to Object 1 in the infant data and selected a 20‐s segment of infant data before and after each onset. Next, we assigned ‘1’ to each frame within the 20‐s segment where the infant was looking at Object 1, and ‘0’ to each frame where there was any other infant look. We repeated this procedure for Object 2 and Object 3. This allowed us to calculate the probability of the infants responding to maternal looks to objects by looking at the *same* object as their mothers. We did the same thing to calculate maternal responsivity to infant looks to objects. Next, we looked at how attention to the partner changed around moments when the mothers/infants looked at the objects. To do so, we selected all mother/infant looks to the objects (irrespective of what object) and followed the same procedure described above in Analysis 3.1. To compare whether the observed responses were significantly higher than chance, we generated control data and followed the same procedure as in Analysis 3.1.

For infants, attention to objects increased both before and after their mothers looked at the objects (Figure 4E). Interestingly, the point at which infants were *most* likely to be looking at the same object as their mothers occurred shortly after the mothers started looking at it. This suggests that although infants often looked at the object before their mothers, their likelihood of focusing on the same object became even higher once their mothers directed their gaze towards it (the CBP test indicated that there was a significant difference between observed and control data, p_5M_ < 0.001, p_15M_ < 0.001; Figure 4E). As a result, attention to their partner decreased around, and especially after, moments where the mothers looked at an object (CBP test, p_5M_ = 0.007, p_15M_ < 0.001; Figure 4F). For mothers, the probability of looking at the same object as their infants increased around the time when infants looked at an object at both ages (CBP test, *p*_5M_ = 0.002, *p*_15M_ < 0.001; Figure 4G), but it was significantly higher at 15 months compared to 5 months (CBP test, *p*_5Mvs15M_ < 0.001; Figure 4G). Interestingly, the probability of mothers looking at their infant increased before infants directed their gaze towards an object and decreased after infant look‐to‐object onset (CBP test, *p*_5M_ = 0.007, *p*_15M_ = 0.005; Figure 4H).

Overall, we did not find evidence to support the hypothesis that mothers' looking behavior have a greater influence on infant's attention over time. However, changes in the infant's looking behaviors associated with mother's attention more strongly at 15 months than at 5 months (Figure 4G).

### 2.4 Analysis 4. The role of partner looks in organising episodes of joint attention

Finally, we asked whether older infants show more intention to involve others (Hypothesis 3). If so, we predicted that intentional ostensive signals such as infant looks to partner *before* and *during* both *leader* and *follower* looks would increase with time. First, in order to understand the use of ostensive cues by the members of the dyad, we explored the probability that a mother/infant would look at their partner *during* as well as *before* any attentional look (Analysis 4.1). Next, we examined whether maternal and infant engagement in ostensive signals such as looks to partner affected the likelihood of looks being followed or not (Analysis 4.2).

#### 2.4.1 Analysis 4.1. Probabilities to Look at the Partner During as Well as Before an Attentional Look

##### 2.4.1.1 Looking at the Partner During an Attentional Look

**FIGURE 5:**
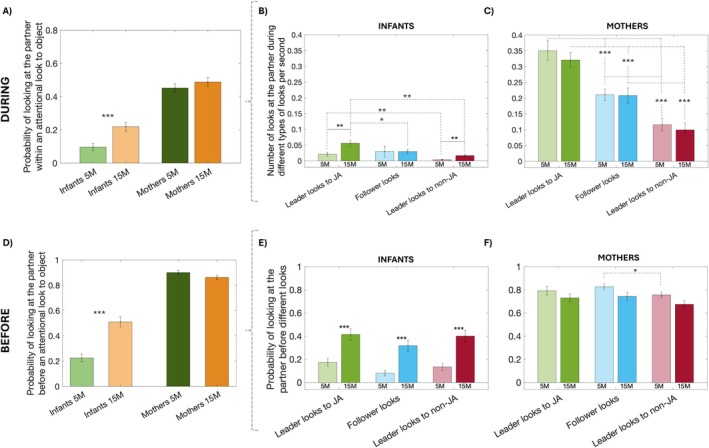
Probability of looking at the partner *during* and *before* initiating an attentional look. (A) Probability of infants (light colors; left) and mothers (dark colors; right) to look at the partner *during* an attentional look to an object. (B and C) Average number of looks (per second) at the partner during leader looks to JA (green), follower looks (blue), and leader looks to non‐JA (red) for infants at 5 months (B; light colors) and 15 months (B; dark colors), and mothers at 5 months (C; light colors) and 15 months (C; dark colors). (D) Probability of infants (light colors; left) and mothers (dark colors; right) to look at their partners *before* any kind of attentional look to an object (i.e., irrespective of whether these looks led to JA and whether they were leader or follower looks). (E) Probability of infants and (F) mothers to look at the partner before leading a look to JA (green), following a look (blue) and leading a look to non‐JA (red). Asterisks indicate significance (*p < 0.05, **p < 0.01, ***p < 0.001). All analyses except A and D are done using interpolated data as it is what allows us to work with leader/ follower looks.

First, we calculated the likelihood that 5‐and 15‐month‐old infants would look at their mothers *during* an attentional episode to an object. To do this, we took the original (non‐interpolated) data, calculated how frequently the infant switched their gaze to their mothers and went back to looking at the same object, and divided it by the total number of times infants looked at an object. We did the same with the data from the mothers. Looking at the partner *during* an attentional episode was infrequent for infants at both ages (Figure 5A). However, older infants were significantly more likely to look to their partners, at least once, during episodes of attention to objects compared to younger infants (*t*(44) = −3.589, *p* < 0.001; Figure 5A). There were no significant differences for mothers (t(44) = −0.989, *p* = 0.328; Figure 5A).

Following this, we computed the number of times per second infants looked at the mothers *during* leader looks to JA, follower looks, and leader looks to non‐JA separately. To do this, we first selected the onsets and offsets from these three types of looks. Next, we went back to the original (non‐interpolated) data and counted the instances when infants looked at the mothers within these onset and offsets (e.g., times when infant led a look to “object 1”, looked at the “parent” and then looked back at “object 1”). To account for the fact that longer looks are more likely to include looks at the partner than shorter looks, we normalized each “look to partner count” by dividing it by the duration of each look (in seconds). Again, looking at the partner during a look was relatively infrequent (Figure 5B), with less than 0.1 looks to the partner per second, irrespective of the type of look. Older infants, however, looked more at their partners than younger infants during leader looks to JA (*t*(44) = −3.137, *p* = 0.003; Figure 5B) and leader looks to non‐JA (*t*(44) = −3.034, *p* = 0.004; Figure 5B). There were no significant differences for mothers (Figure 5C; refer to Table S4 for more details on the results).

Interestingly, there were significant differences between the average number of looks to the partner *during* the three different types of looks. At both timepoints, infants performed more looks to the partner during leader looks to JA compared to leader looks to non‐JA. Older infants, but not younger infants, also performed more looks to the partner during leader looks to JA compared to follower looks (Figure 5B; refer to Table S4 for more details on the results). A similar pattern was observed for mothers. At both ages, they performed more looks to the partner during leader looks to JA than any other type of look. Looks to the partner during leader looks to non‐JA were the least frequent (Figure 5C; refer to Table S4 for more details on the results).

##### 2.4.1.2 Looking at the Partner Before Initiating an Attentional Look

Next, we explored the likelihood of looking at the partner *before* initiating a look at an object (Figure 5D–F). First, we looked at whether infants looked towards their mothers before *any* kind of attentional look. To do this, we took the interpolated data and calculated the proportion of times any infant look at the object was preceded by a look at the partner. We found that older infants were more likely to look at their parents right before any type of attentional look than younger infants (*t*(44) = −5.535, *p* < 0.001; Figure 5D). We performed the same analyses using the data from the mothers and found no significant differences (*t*(44) = 1.686, *p* = 0.099; Figure 5D).

Next, we looked at whether the likelihood of looking towards the partner *before* an attentional look differed between leader looks to JA, follower looks, and leader looks to non‐JA (Figure 5E,F). We found that older infants were consistently more likely to look at their parents before leading a look to JA (*t*(44) = −4.081, *p* < 0.001), following a look (*t*(44) = −4.353, *p* < 0.001) and leading a look to non‐JA (*t*(44) = −4.528, *p* < 0.001) than younger infants (Figure 5E). There were no significant differences within age groups between the different types of looks (Figure 5E; refer to Table S5 for more details on the results). The same was true for the mothers, with the exception that, at 5 months, mothers were more likely to look at the partner before a follower look compared to a leader look to non‐JA (Figure 5F; refer to Table S5 for more details on the results).

Overall, looking at the partner *during* and *before* an attentional episode was infrequent for infants at both ages. However, older infants used these ostensive signals more often than younger infants. At both ages, looks to the mothers *during* attentional episodes were more frequent when infants led their partners into JA than when they did not, possibly to ensure their gaze was being followed. In contrast, looks *before* attentional episodes appeared more indiscriminate, with no significant differences between leader looks to JA and to non‐JA.

#### 2.4.2 Analysis 4.2. Likelihood of Following Leader Looks That Are Preceded by Partner Looks

Finally, we examined whether maternal and infant engagement in ostensive signals such as looks to partner affected the likelihood of looks being followed or not. We reasoned that if ostensive signals contribute to infants becoming better at leading, we should observe stronger effects of infant ostensive behaviors on mothers over developmental time. Alternatively, if ostensive signals contribute to infants becoming better at following, we should observe stronger effects of maternal ostensive signals on infants.

To do so, we first took all infant leader looks (i.e., leader looks to JA and leader looks to non‐JA) and split them into two groups: the ones that were preceded by a partner look and the ones that were not. Next, we took these two types of looks and examined whether mothers followed them or not. To do so, we took each infant look, time‐locked it to the mother's data and checked whether in the 5‐s following that look, the mother followed (i.e., the mother looked at the same thing as the infant) or not. If a look was followed, we assigned “1”, if it was not, we assigned “0”. This allowed us to create a time series for the mothers' responses. To test for significant differences, we employed a CBP test (see Section 1.3.3). Of note, previous research has also used a 5‐s window to check for follower looks (see Northrup and Iverson 2020; Yu and Smith 2013).

**FIGURE 6:**
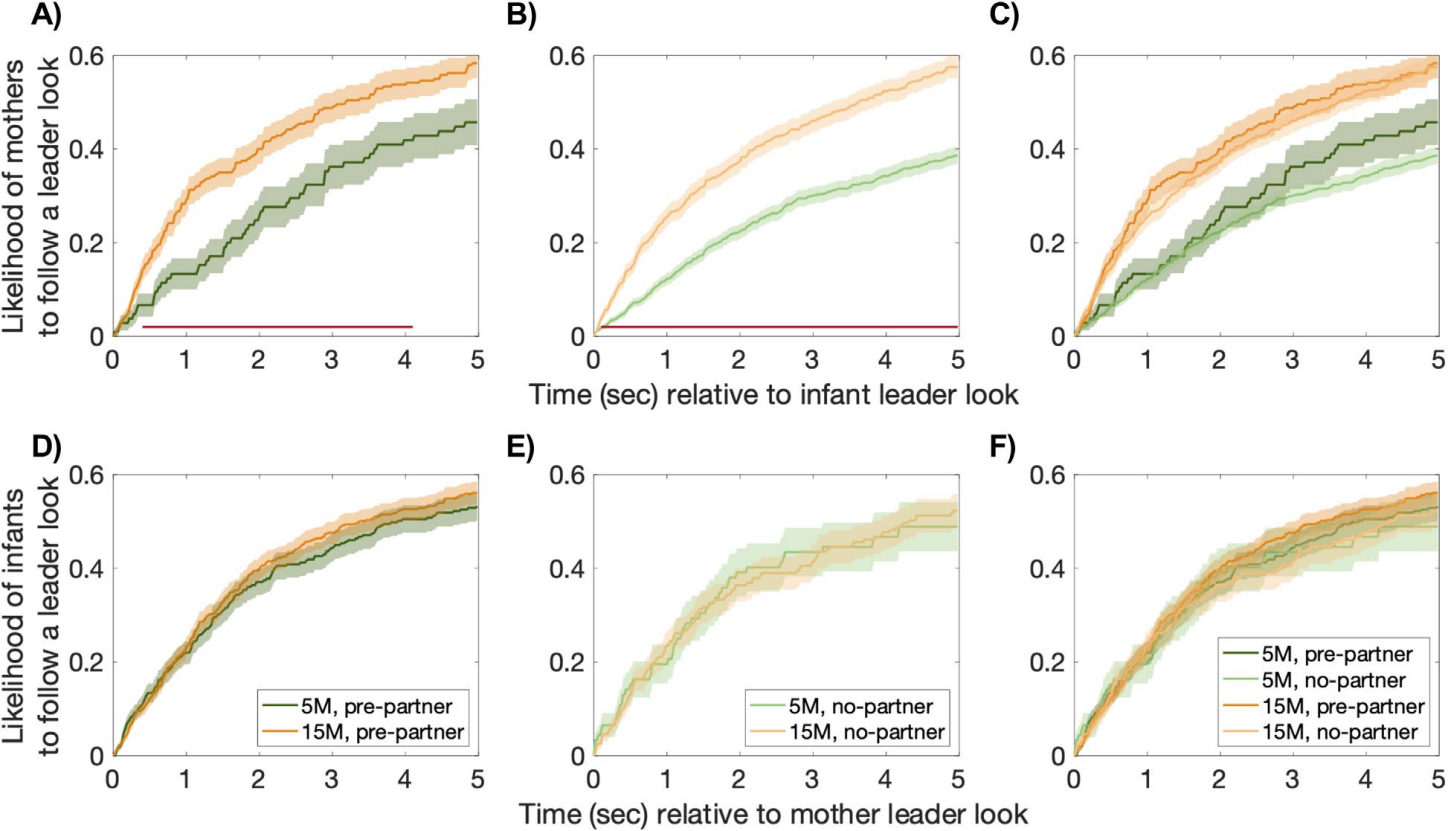
Likelihood of following leader looks that are preceded by partner looks. Likelihood of mothers (A–C) and infants (D–F) to follow looks that are preceded by a partner look (dark colors; *pre‐partner*) (A and D) versus looks that are not preceded by a partner look (light colors; *no‐partner*) (B and E). In green is the data at 5 months and in orange at 15 months. (A, B, D and E) show differences across age groups. (C and F) show differences within age groups (i.e., looks preceded by partner looks vs. looks not preceded by partner looks). Red thick lines indicate significance from the CBP test (*p* < 0.025, two‐sided).

There were no differences in the likelihood of mothers following an infant leader look that was preceded by a partner look compared to an infant leader look that was not preceded by a partner look, at both timepoints (Figure 6C). However, at 15 months, mothers were in general more likely to follow an infant leader look compared to 5 months, regardless of whether it was preceded by a partner look (Figure 6A,B).

Next, we compared the likelihood that infants would follow a mother look that was preceded by a partner look. There were no differences across time points (Figure 6D,E), and the likelihood of infants following a mother leader look did not change based on whether these looks were preceded by a partner look or not (Figure 6C).

Together, these findings suggest that the use of ostensive signals, such as looks to the parent, has little effect on infants and their mothers, and it does not seem to change from 5 to 15 months. However, it is worth noting that at 15 months, mothers are more likely to follow their infants than at 5 months. This suggests that something in the infant behavior, other than looks directed towards the partner, might be increasing their intentionality and making them easier to be followed.

## 3 Discussion

This study investigated the mechanisms that drive JA and how these change between 5‐ and 15‐month‐old infants. First, we explored changes in real‐world JA and asked whether these are driven by infants becoming better at leading their partner (Hypothesis 1) or at following their partner (Hypothesis 2) into JA. Next, we examined whether changes in JA are facilitated by development in intentionally mediated forms of communication (Hypothesis 3). To do so, we observed free‐flowing tabletop toy play between *N* = 48 infants at 5 and 15 months and their mothers, and micro‐coded their gaze behaviors at 50fps. By tracking the momentary visual fixations of each participant, we measured how often they attended to the same object at the same time and how they entered and organized these JA episodes. Across all our analyses, we also monitored the mother's behavior to examine whether observed changes are due to changes solely in the infant, or whether changes in parent behavior also play a role.

During infancy, mothers switched attention rapidly between play objects and the infant's face. This pattern did not change between 5 and 15 months. In contrast, 5‐month‐old infants looked only occasionally towards the mother's face. As infants grew, these dynamics changed. Older infants shifted attention more often towards their partner (Figure 2A), but no equivalent changes were observed in mothers' behavior (Figure 2A). Figure 2A is also important as it provides evidence that 5‐month‐old infants were as proficient as 15‐month‐olds in alternating gaze within this quasi‐naturalistic setup, generating a comparable number of opportunities for JA as their older counterparts.

At both time points, most of the JA moments were driven by the mothers responding to the infants' initiations of attention rather than leading the infants' attention (of all maternal looks that led to JA, 67% were follower looks at 5 M and 63% at 15 M; Figure 3A, blue vs. green bars). These findings are in line with previous research (Evans and Porter 2009; Mendive et al. 2013; Phillips 2023; Yu and Smith 2013) and suggest that, during infancy, the mothers' role is mostly in monitoring and contingently responding to reorientations in their infants' gaze. In following the focus of their infants' attention at moments when they reorient towards a new object, the mother “catches” and extends infant attention with reactive and dynamic change in their salient ostensive behaviors, to which infants are responsive (Figure 4; Phillips 2023; Suarez‐Rivera et al. 2019; Wass, Clackson, et al. 2018).

Importantly, though, our results suggested that the frequency of mothers' follower looks did not change significantly between 5 and 15 months. However, what did change significantly over this period was a reduction in the likelihood that a new attention episode initiated by the infant would fail to result in JA (i.e., leader looks to non‐JA decreased with age; Figure 3A, maroon bars). This drives our overall finding that episodes of JA increased in duration and frequency (Figure 3B,C). Next, we discuss potential factors that could have contributed to these changes in JA.

First, we hypothesized that infants would become better at leading their partners into JA (Hypothesis 1). Consistent with this, we observed an increase in infant leader looks that led to JA as opposed to leader looks that did not lead to JA over time (Figure 3A). Here, for the first time, we also observed changes in the mothers' behavior—namely that mothers with older, but not younger, infants responded when infants looked at them by looking back at their infants (Figure 4D) and were also more likely to respond to infants' looks to objects by looking at the same object as their infants (Figure 4G). Similarly, we also observed that mothers were more likely to follow an infant leader look at 15 months compared to 5 months (Figure 6A,B). Taken together, these findings fit well with the idea that JA is increasingly infant‐driven (Hypothesis 1). Consistent with our predictions, older infants did not only produce more leader looks that led to JA than younger infants but they also appeared to have a greater influence on their mothers' attention.

As an alternative hypothesis, we hypothesized that infants would become better at following their partners into JA (Hypothesis 2). At both ages, infants were responsive to their partners' behaviors (Figure 4). For example, although infants frequently looked at the object before their mother, the likelihood of infants looking at the same object as their mother peaked immediately after the mother began looking at it (Figure 4E). This indicates that infants became even more likely to focus on an object after noticing their mother was looking at it (Figure 4E). However, contrary to our predictions, we found no evidence of older infants showing greater responsiveness to their mothers' looking behavior than younger infants (Figure 4B,E). The proportion of follower looks, however, increased over time (Figure 3A). This is in line with similar other studies (Kochanska and Aksan 2004; Scaife and Bruner 1974) and suggests that older infants are more capable of adjusting their own looking behavior in response to their partner's behavior. Overall, our findings seem to support both hypotheses: older infants became better at leading and following their partners into JA, demonstrating that these two abilities are complementary aspects of their developmental progress.

Finally, we examined whether these changes in leading and following were facilitated by developments in intentionally mediated forms of communication (Hypothesis 3). First, we discuss our findings on how infants used ostensive signals both during and before leading a look. At both ages, infant looks to the partner during infant leader looks to JA were more frequent than during leader looks to non‐JA (Figure 5B). Similar to other research (Phillips et al. 2023), this suggests that infants are aware of whether their attention is being followed and perform more *“checking looks”* when it is, possibly to ensure their gaze is indeed being followed. This finding also suggests that processes important to triadic engagement such as looks to the parent's face may already be coming online at this younger age. This is striking as research using more structured paradigms has suggested that infants typically do not exhibit these behaviors until they reach 9 months of age (e.g., Carpenter et al. 1998). Despite this, we observed that the probability of infants looking at the partner at least once during an attentional look to an object was higher for older infants compared to younger infants (Figure 5A). These findings might indicate not only the increased ability of older infants to control their attention and shift between person and object, but also that older infants check more often whether their attention is being followed than younger infants. Similarly, older infants were more likely to look at their partners *before* leading a look (Figure 5D–E). When we repeated the same analyses to examine developmental changes in mothers' behavior, we observed no significant changes between 5 and 15 months (Figure 5A,C,D,F).

Some researchers have interpreted these shifts in infant gaze between the partner and the goal (i.e., the toy) as evidence that infants' behaviors become increasingly intentional and communicative (Carpenter et al. 1998; Tomasello and Carpenter 2006). An interpretation like this fits well with the fact that infants became better leaders and that mothers with older, but not younger infants, were more likely to follow their infants' leader looks (Figure 6A,B). In line with active learning theories, over time, infants seemed to improve in signaling their intentions, leading to a greater influence on their mother's attention. Some aspects of our findings, however, are harder to reconcile with this conclusion. That is, even at 15 months, infants did not perform many “checking partner looks” *during* leading a look to verify whether their partner followed their attention (i.e., the average number of looks per second at the partner during leader looks to JA was lower than 0.1; Figure 5B). Consequently, these more frequent (though still relatively infrequent) “partner checking looks” appear to be a plausible factor, although not the only one, influencing infants' increased ability to lead their parents' attentional interest and possibly reflect a growing (but not fully developed) understanding of others as intentional beings. Similarly, it is important to highlight that the proportion of infants' looks to the parent's face *before* leading a JA episode was relatively small (~0.2 at 5 months and ~0.4 at 15 months; Figure 5E) and thus, these looks, at best, can explain only a small proportion of the observed leading looks to JA episodes.

Finally, there are two more reasons to believe that infant ostensive signals, such as looks to the partner, are not the primary factor driving changes in their partners' behavior. First, we found no differences in the frequency of infant looks to the partner before leading a look to JA compared to leading a look to non‐JA (Figure 5E). Second, the likelihood of mothers following an infant's leader look was not affected by whether it was preceded by a look to the partner (Figure 6C). Ultimately, other communicative behaviors coming from the infant such as vocalizations (Carpenter et al. 1998; Liszkowski and Tomasello 2011), pointing (Begus and Southgate 2012; Liszkowski and Tomasello 2011), or other hand actions (e.g., object manipulation; Yu and Smith 2013) might also play a role in establishing infant‐led JA.

As described above, we observed overall relatively few changes in mothers' behavior across the same developmental time interval. For example, we did not observe changes over time in the proportion of instances where mothers either followed or led infants into JA (Figure 3A). Despite this lack of overt behavioral changes, it remains possible however that over time, mothers might have become better at perceiving and understanding their infant's signals and following their lead more effectively. An explanation like this would fit with some of our findings (Figures 4D,G and 6A,B) and could partly explain the increase in infant‐led JA we observed.

Lastly, we discuss our findings on how infants use ostensive signals to follow their partner's looks. We found no differences between older and younger infants in the likelihood of shifting their gaze towards their parents *during* follower looks (Figure 5B). At 15 months, however, infants were more likely to look at their parents *before* following a look compared to 5‐month‐old infants (Figure 5D–E). In line with the theory of natural pedagogy (Csibra and Gergely 2009, 2011), this behavior might have helped them verify where their parents were focusing on and follow them more accurately, making them better at following. Despite these age differences, however, it is worth noting that the proportion of infants' looks to the parent's face at 15 months *before* following a JA episode was, again, relatively small (< 0.5) so the increase in infant follower looks might only be partly explained by an increase in infant intentionally mediated behaviors. This fits well with previous research (e.g., Yu and Smith 2013, 2017; Custode and Tamis‐LeMonda 2020), that has found no evidence that looking at their partners' face plays any role in guiding infants' follower looks to JA. One explanation for this might be that infants are more likely to follow the hand actions (e.g., object manipulation) of their partners rather than the direction of their gaze to coordinate visual attention with them (Yu and Smith 2013). This provides not only a faster, but also a more spatially precise pathway into infant‐followed JA (Yu and Smith 2013, 2017). Alternatively, it is possible that mothers, observing that older infants tend to be more inattentive (Figure 2B), chose to make themselves more salient (e.g., by increasing the rate of modulation of the voice or engaging in object‐related talks) as a more efficient way to direct and maintain the infants' attention to certain objects than looks to the partner (Phillips et al. 2023). In line with this, we found that the likelihood of infants following a mother's leader look was not affected by whether it was preceded by a look to the partner (Figure 6D–F). Collectively, contrary to our hypothesis 3, our findings suggest that the use of ‘looks to partner’ has little effect on infants and their mothers, and it does not seem to help organize episodes of JA.

Overall, the current study showed that, with time, the initiations of JA became more equally distributed within the members of the dyad. This shift evolved from the infants being passively engaged in JA by the mothers to the infants actively following and engaging the mothers some months later. As a result, older infants not only became more efficient leaders (i.e., performed more leader looks that led to JA, and changes in infants' looking behavior had a greater influence on mother's attention, Hypothesis 1) but also followers (i.e., engaged in more follower looks to JA, Hypothesis 2). Older infants also appeared more intentional (i.e., performed more looks to the partner) (Hypothesis 3); nevertheless, even at 15 months, JA was still predominantly achieved through mechanisms other than looking towards the partner.

In both scenarios, improved leading and following were predicted to lead to a greater chance of infants and mothers coordinating their attention with each other. This was evident in JA episodes becoming longer and more frequent.

Understanding differences in establishing JA and how these change over developmental time is an important goal, not only because differences in the frequency of use of JA behaviors are related to subsequent language, cognitive, and social development in typical samples, but also because it may offer insights into the underlying nature of various conditions, such as autism and attention‐deficit/hyperactivity disorder, where impairments in establishing JA are frequently observed (Brooks and Meltzoff 2005; Mundy et al. 2007). Consequently, understanding the emergence of JA in more naturalistic scenarios seems crucial for identifying potential signs of atypical development at an early stage, allowing for timely intervention and support.

### 3.1 Limitations

Our findings should be interpreted with consideration to a number of limitations of the study. First, we focused exclusively on overt (visual) attention and specifically examined looking behaviors of both infants and their mothers. However, we know that visual attention is not the only modality that can influence JA and that there are many other behaviors that can shape these dyadic processes through which JA is established such as vocalizations, gestures (e.g., pointing, object handling) and touch (amongst others) (Yu and Smith 2017; Schroer and Yu 2022; Suarez‐Rivera et al. 2019; Deák, Flom and Pick 2000). Second, observing mother‐infant dyads interact in a free play setting within a table‐top interaction provided a more naturalistic setting and increased generalizability of the study. However, using only this free play task may have limited the variability of interactional patterns that the dyads engaged in. It may have also put pressure on mothers to engage in the interaction more than they would have otherwise, since they were given directions to “play with their children as they normally would” while being video recorded (Abney et al. 2020). Third, our cross‐sectional design limited our ability to make predictions regarding the stability and predictability of specific dyadic dynamics. It is likely that certain characteristics of the dyad (e.g., mother or infant over‐ or under‐responsiveness) may, to an extent, influence the way JA is organized and how it changes over time (Evans and Porter 2009). Relatedly, we know that the history of interactions with others such as siblings, peers, and other caregivers, may also shape the way infants and mothers interact and establish JA. However, in this study we did not consider the potential varying levels of exposure to others nor its impact on the dynamics between infants and their mothers. Fourth, our sample is considerably homogeneous in terms of ethnicity, culture and socioeconomics and consists only of mothers (Table S1). It will be useful for future studies to include investigations from more heterogeneous groups/ communities (Taverna et al. 2024; Feldman 2007; Mundy et al. 2007) as well as to include fathers, given their increased involvement in their infants' lives. Finally, throughout the discussion we considered this “checking behavior” from the infant to the parent as reflecting some kind of monitoring of the partners' behaviors. Nonetheless, alternative interpretations have been proposed by other researchers. For example, it could be that that these behaviors from the infants might be merely to verify their mother's presence (e.g., for emotion regulation, see Carpenter et al. 1998) or just displaying conditioned responses to their mothers' smiles, contingent vocalizations, and other expressions of pleasure and interest. Interpretations like these would indicate that infants do something different than monitoring the adults' intentional behavior.

## Supporting information


Supporting Information S1.

Publication‐Checklist‐Form.

## Data Availability

Partial restrictions to the data and/or materials apply. Due to the personally identifiable nature of this data (video and audio recordings from infants and their mothers) the raw data will not be made publicly accessible. Researchers who wish to access the raw data should email the lead author, and permission to access the raw data will be granted as long as the applicant can guarantee that certain privacy guidelines can be provided. The analytic code necessary to reproduce the analyses presented in this paper is not publicly accessible but can be shared upon request. The analyses presented here were not preregistered.
