# Supplementary material for: Who Leads and Who Follows? The Pathways to Joint Attention During Free‐Flowing Interactions Change Over Developmental Time
Source: Child Dev. 2025 Feb 18;96(3):1112–27. doi: 10.1111/cdev.14229 (PMC12023812; doi:10.1111/cdev.14229)
Supplement: Supplementary file 1 — Supporting Information S1. [file CDEV-96-1112-s001.docx]

**Supplementary materials:**

1. Table S1. Demographic data at 5 and 15 months.
2. Table S2. Table summarising the numbers of datasets included in the analyses for both samples as well as reason for exclusion.
3. Figure S1. Toys employed at both time points.
4. Figure S2. Example of infant raw data.
5. Figure S3. Sensitivity analysis using a different threshold for interpolation.
6. Table S3. Description of different looks of interest.
7. Table S4. Results on the likelihood to look at the partner *during* an attentional episode to an object.
8. Table S5. Results on the likelihood to look at the partner *before* an attentional episode to an object.
9. Figure S4 Average number of looks per minute.
10. Table S6 and Table S7. Results and specific models employed for the LME analyses on probability of mothers to follow infant leader looks.

**Table S1.** Demographic data at 5 and 15 months.

|  | **5 months** | **15 months** |
| --- | --- | --- |
| Maternal education | % | % |
| Post-graduate degree | 53.33 | 47.06 |
| Degree | 40 | 35.29 |
| F.E. Qualification | - | - |
| A Level | 6.67 | 11.76 |
| GCSE's | - | 5.88 |
| No formal qualification | - | - |
| Other | - | - |
| Prefer not to answer | - | - |
| Household income | % | % |
| Under 16k | - | - |
| 16k - 25k | - | - |
| 26k - 35k | - | - |
| 36k - 50k | 6.67 | 5.88 |
| 51k - 80k | 20 | 17.65 |
| More than 80k | 60 | 58.82 |
| Prefer not to answer | 13.33 | 17.65 |
| Maternal ethnicity | % | % |
| White British | 60 | 64.71 |
| Other White | 13.33 | 5.88 |
| Asian, Indian | 6.67 | 11.76 |
| Black | 13.33 | 5.88 |
| Mixed – White/Afro-Caribbean | - | - |
| Not answered | 6.67 | 11.76 |

**Table S2.** Table summarising the numbers of datasets included in the analyses for both samples as well as reason for exclusion.

| Final datasets | 5 months | 15 months |
| --- | --- | --- |
| Gaze coded (N) | 47 | 48 |
| Infants | 24 | 24 |
| Mothers | 23 | 24 |
| Dyads incomplete * | 1 | 2 |
| Dyads completed | **23** | **23** |

* The data for one of the members of the dyad (infant or mother) could not be recovered (i.e. datasets were either corrupted or missing).

**Toys**
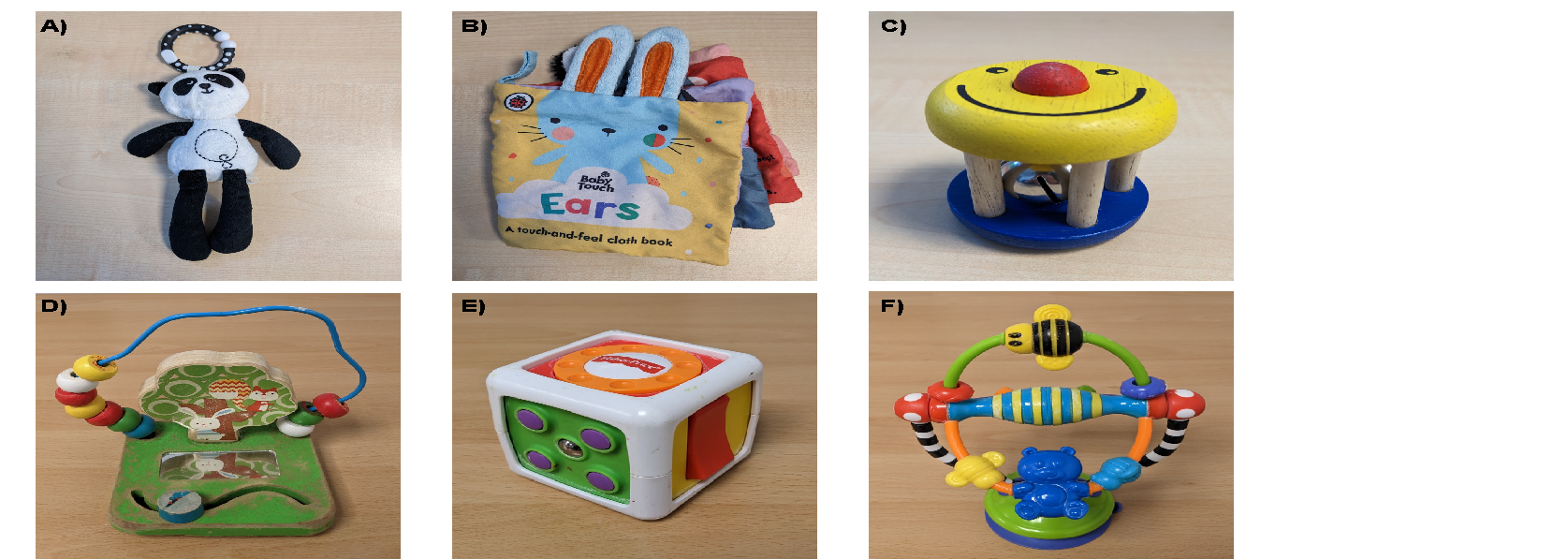


**Figure S1.** Photos of the toys employed at both time points: panda (A), a book (B) and a rattle (C).


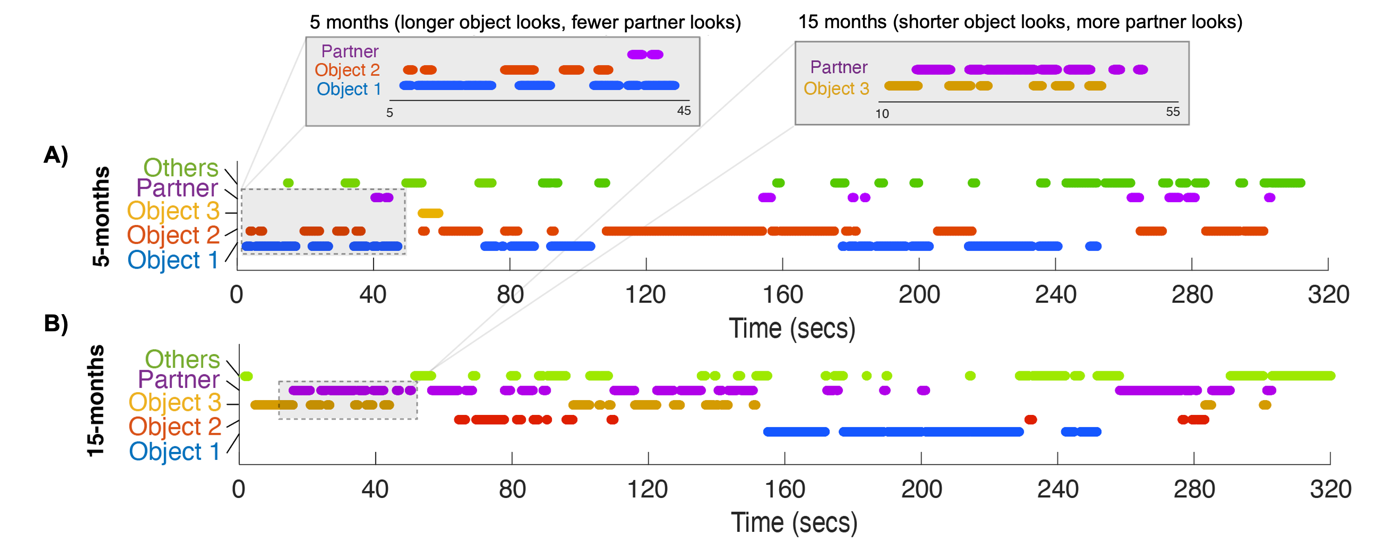
**Figure S2.** Example of infant raw data. This figure shows the time series data of two infant participants at 5 (A) and 15 months (B). Looks of interest (i.e. looks towards the three objects and to partner) are plotted separately, one in a different row and colour. The rest of the looks, i.e. inattentive looks and uncodable moments, are grouped together and named as 'others’. In this example we can see the differences in behaviour at 5 versus 15 months. At 5 months, infants performed slightly longer and more looks to objects with fewer partner looks. Instead, at 15 months, infants performed more looks to their mothers and the looks to objects were generally shorter.

**Figure S3. Sensitivity analysis using a different threshold for interpolation.** This figure presents the main results in Analysis 2 using a threshold for interpolation of 300ms instead of 2sec. This was done to confirm the consistency of our findings and make sure that the relatively arbitrary threshold of 2 seconds was influencing our main findings. (A) shows the proportion of leader looks to non-JA (in red), leader looks to JA (in green) and follower looks to JA (in blue). Infant (left) and mother (right). (B) Shows the average duration of JA and (C) shows the average number of JA episodes per minute. Asterisks indicate significance (* = p<0.05, ** = p<0.01, *** = p< 0.001).

Similar to Figure 3, we observe that at 15 months, infant’s initiations are more likely to lead to JA with their partner compared to 5 months (i.e. they perform more leader looks to JA and less leader looks to non-JA) and they engage in more follower looks (Fig S2A). Consequently, similar to Figure 3, JA episodes become longer and more frequent (Fig S2B and C).

**Table S3.** Definition of different types of looks and attention episode categories.

| **Looks to objects** | | Participant is looking at one of the three objects (panda, rattle, book) |
| --- | --- | --- |
| **Looks to partner** | | Participant is looking at the partner's face |
| **Others** | **Inattentive** | Participant is not looking to any of the objects nor to the partner's face |
|  | **Uncodable** | The view of the participant’s eyes is blocked |
|  |  |  |
| **Leader look** | **Leader look to non-Joint Attention** | Participant (e.g. infant) shifted their gaze towards an object that the partner (e.g. mother) was not already looking at, and the partner (e.g. mother) did not follow the look towards the object at any point during the time that the participant (e.g. infant) was still attenting towards the object |
|  | **Leader look to Joint Attention** | Participant (e.g. infant) shifted their gaze towards an object that the partner (e.g. mother) was not already looking at, and the partner (e.g. mother) followed their look towards the object that the participant (e.g. infant) was attenting to. |
| **Follower look** | | Participant (e.g. infant) shifted their gace towards the same object that the partner was attending to. |
|  |  |  |
| **Joint Attention** | | Periods of time when both partners were looking at the same object at the same time |

Table S4. Results on the likelihood to look at the partner *during* an attentional episode to an object.

|  | INFANTS | | |  | MOTHERS | | |
| --- | --- | --- | --- | --- | --- | --- | --- |
| **Differences between age groups (5M vs. 15M)** | *p_value* | *t stat* | *df* |  | *p_value* | *t stat* | *df* |
| Leader looks to JA | 0.003 | -3.137 | 44 |  | 0.466 | 0.735 | 43 |
| Follower looks | 0.988 | 0.015 | 42 |  | 0.938 | 0.079 | 44 |
| Leader looks to non-JA | 0.004 | -3.034 | 44 |  | 0.579 | 0.558 | 44 |
|  |  |  |  |  |  |  |  |
| **Differences at 5M** | *p_value* | *t stat* | *df* |  | *p_value* | *t stat* | *df* |
| Leader looks to JA vs. Follower looks | 0.749 | -0.325 | 20 |  | 0 | 4.609 | 21 |
| Leader looks to JA vs. Leader looks to non-JA | 0.01 | 2.84 | 22 |  | 0 | 7.045 | 21 |
| Follower looks vs. Leader looks to non-JA | 0.182 | 1.384 | 20 |  | 0.001 | 3.813 | 22 |
|  |  |  |  |  |  |  |  |
| **Differences at 15M** | *p_value* | *t stat* | *df* |  | *p_value* | *t stat* | *df* |
| Leader looks to JA vs. Follower looks | 0.002 | 3.575 | 22 |  | 0 | 8.259 | 22 |
| Leader looks to JA vs. Leader looks to non-JA | 0.001 | 4.075 | 22 |  | 0 | 13.628 | 22 |
| Follower looks vs. Leader looks to non-JA | 0.055 | 2.023 | 22 |  | 0 | 5.436 | 22 |

Table S5. Results on the likelihood to look at the partner *before* an attentional episode to an object.

|  | INFANTS | | |  | MOTHERS | | |
| --- | --- | --- | --- | --- | --- | --- | --- |
| **Differences between age groups (5M vs. 15M)** | *p_value* | *t stat* | *df* |  | *p_value* | *t stat* | *df* |
| Leader looks to JA | 0 | -4.081 | 44 |  | 0.234 | 1.207 | 43 |
| Follower looks | 0 | -4.354 | 42 |  | 0.053 | 1.987 | 44 |
| Leader looks to non-JA | 0 | -4.528 | 44 |  | 0.057 | 1.955 | 44 |
|  |  |  |  |  |  |  |  |
| **Differences at 5M** | *p_value* | *t stat* | *df* |  | *p_value* | *t stat* | *df* |
| Leader looks to JA vs. Follower looks | 0.075 | 1.878 | 20 |  | 0.267 | -1.141 | 21 |
| Leader looks to JA vs. Leader looks to non-JA | 0.245 | 1.194 | 22 |  | 0.436 | 0.794 | 21 |
| Follower looks vs. Leader looks to non-JA | 0.128 | -1.588 | 20 |  | 0.042 | 2.154 | 22 |
|  |  |  |  |  |  |  |  |
| **Differences at 15M** | *p_value* | *t stat* | *df* |  | *p_value* | *t stat* | *df* |
| Leader looks to JA vs. Follower looks | 0.064 | 1.951 | 22 |  | 0.79 | -0.269 | 22 |
| Leader looks to JA vs. Leader looks to non-JA | 0.805 | 0.25 | 22 |  | 0.099 | 1.724 | 22 |
| Follower looks vs. Leader looks to non-JA | 0.1 | -1.717 | 22 |  | 0.063 | 1.957 | 22 |

**Figure S4. Average number of looks per minute.** Leader look to non-JA (in red), leader looks to JA (in green) and follower looks to JA (in blue). Infant (left) and mother (right).

**Results and specific models employed for the LME analyses on probability of mothers to follow infant leader looks.**

**Table S4.** Model for probability of mothers following an infant leader look as a function of a leader look following a look to partner or not.

| Dependent/ Response variable = probability of following an infant leader look at 5M (Model 1) or 15M (Model 2)  Fixed effects (predictor variable) = Leader look that follows a partner look vs leader look that did not  Random effect for subject | | | | | |
| --- | --- | --- | --- | --- | --- |
| **Model 1 - 5 months** | | | | | |
| Time window | ß value | Std.Error | DF * | t-value | p-value |
| 2 min | -0.03 | 0.02 | 40 | -1.37 | 0.18 |
| 5 min | -0.03 | 0.02 | 40 | -1.58 | 0.12 |
| 10 min | -0.02 | 0.02 | 40 | -1.43 | 0.16 |
| **Model 2 - 15 months** | | | | | |
| Time window | ß value | Std.Error | DF | t-value | p-value |
| 2 min | -0.10 | 0.02 | 44 | -3.86 | 0.00 |
| 5 min | -0.09 | 0.02 | 44 | -4.71 | 0.00 |
| 10 min | -0.08 | 0.01 | 44 | -6.25 | 0.00 |

* Subjects with only one look were excluded from the analyses

**Table S5.** Model for probability of mothers following an infant leader look as a function of age.

| Dependent/ Response variable = probability of following a leader look that is preceded by a partner look  Fixed effects (predictor variable) = Age group  Random effect for subject | | | | |
| --- | --- | --- | --- | --- |
| **Time window post look = 2sec** | | | | |
| ß value | Std.Error | DF | t-value | p-value |
| 0.14 | 0.03 | 40 | 4.17 | 0.00 |
| **Time window post look = 5sec** | | | | |
| ß value | Std.Error | DF | t-value | p-value |
| 0.13 | 0.03 | 40 | 4.81 | 0.00 |
| **Time window post look = 10sec** | | | | |
| ß value | Std.Error | DF | t-value | p-value |
| 0.12 | 0.03 | 40 | 4.64 | 0.00 |
