# Supplementary material for: Who Leads and Who Follows? The Pathways to Joint Attention During Free‐Flowing Interactions Change Over Developmental Time
Source: Child Dev. 2025 Feb 18;96(3):1112–27. doi: 10.1111/cdev.14229 (PMC12023812; doi:10.1111/cdev.14229)
Supplement: Supplementary file 2 — Publication‐Checklist‐Form. [file CDEV-96-1112-s002.pdf]

Child Development Checklist

Please complete and return. Failure to adhere to manuscript style and publication requirements listed below will result in your manuscript being returned to you for editing. Failure to complete any production steps below may result in delayed publication of your manuscript. Note, completion of forms does not guarantee acceptance/publication; however, acceptance is contingent on compliance with journal policy & requirements.

Corresponding author name:

Manuscript number:

MANUSCRIPT STRUCTURE

✔ 1) ***Manuscript***

a) Is no longer than **40-45 pages** (papers over 45 pages will be asked to revise for length)
b) Has **1-inch margins**
c) Is **double-spaced** (including *abstract, body text and references*)
d) Uses **12-pt. Times New Roman** font

✔ 2) ***Running Head*** is supplied

✔ 3) ***Abstract***

a) Is **120 words or fewer**
b) Includes participants' **numerical age, sex/gender, and race/ethnicity, as well as years that assessment(s) took place**
c) **Total number of participants** (Ns) is included
d) **Is written in the third person**, not first person
e) Includes a **characterization of the focal effect size**, if applicable (*generally as a* ***d*** *or* ***r*** *for quantitative studies).*

✔ 4) ***Sections***

a) *Empirical articles* must have the following sections: **INTRODUCTION** (***not*** *labeled*), **METHOD**, **RESULTS**, and **DISCUSSION** (***all*** *labeled*).
b) **METHOD** section includes **demographic information of participants**, such as *sex, SES, race or ethnicity, recruitment method*, etc.

✔ 5) ***References***

e) Do not exceed **8 double-spaced pages** (rare exceptions may be made)
f) Are cited both **in text and in the reference list**
g) Are listed in **alphabetical order by authors' surnames** (check to ensure compliance)
h) Contain only the **volume #**, *omitting the issue #*
i) **Include the DOI #** where available, *as required by the APA 7th Edition*

☑ 6) ***Author acknowledgments/notes***

a) All funding sources (if any) for the research reported are listed in the **author notes**
b) Acknowledgments **do <u>not</u> include thanks to action editor**

☑ 7) **No footnotes or endnotes** are used

COMPLIANCE WITH *CHILD DEVELOPMENT* PUBLICATION STANDARDS

To proceed to publication, SRCD requires compliance with its Sociocultural Policy.

☑ 8) The manuscript states the **dates of data collection** (if applicable).

This *information can be found* in the (please check all that apply):
☐ Abstract and/or in the ☑ Methods

Please enter *the page number(s) where dates of data collection can be found* in the Methods: 9

☑ 9) The manuscript states **the theoretically relevant characteristics of the particular sample studied**, *for example, but not limited to race/ethnicity, socioeconomic status, language, sexual orientation, gender identity (inclusive of non-binary options), religion, generation, family characteristics***.**

Demographic characteristics of the sample can be found in the (please check all that apply):
☐ Abstract and/or in the ☑ Methods

Please enter *the page number(s) where dates of data collection can be found* in the Methods: and Table

☑ 10) The manuscript states **the place(s) from which that sample was drawn**, *including country, region, city, neighborhood, school, etc. and all other context variables that are relevant to the focus of the publication, except when it violates expectations of privacy and confidentiality by an institutional review board or the setting itself.*

The place from which participants were drawn can be found in (please check all that apply):
☐ Abstract and/or in the ☑ Methods

Please enter *the page number(s) where the place from which participants were drawn can be found* in the Methods: 9

☑ 11) **If compliance with SRCDs sociocultural policy is not possible** (as reflected in any or all of items 8-10 above), this should be addressed in the manuscript. *If your manuscript does not comply, please indicate the page number in the accepted manuscript where reasons for non-compliance can be found.*

____

☑ 12) **Confirmatory/Exploratory statement** is included in the manuscript, and the cover letter notes the page number where statement appears. Statement addresses *to what degree the analyses presented*

represent a relatively exploratory versus confirmatory effort, & in what ways. See submission guidelines for details: https://bit.ly/3zJMWnT

Please indicate *the page number(s) in the accepted manuscript where the Confirmatory/Exploratory statement can be found.* 7-8

Post-Publication Data, Code, and Materials Availability and Statement of Preregistration

All manuscripts submitted to *Child Development* must be in compliance with the SRCD's *Author Guidelines on Scientific Integrity and Openness in Child Development* (https://www.srcd.org/research/journals/child-development/author-guidelines-scientific-integrity-and-openness-child-development). Consistent with this SRCD Policy, *Child Development* further requires that the following information be provided in the **Acknowledgments section** of all manuscripts submitted and ultimately published in the journal. Note that this information will not be used as part of the review process in that ultimate acceptance/rejection of a submitted manuscript is not conditional on the answers to the following questions. However, as a separate matter, action editors may request data and/or code as part of the review process (without the expectation that those will be made available post-publication).

13) First, please confirm that one of the following is indicated in the Acknowledgements regarding **data availability**:

☐ "The data necessary to reproduce the analyses presented here are not publicly accessible."
OR
☑ "The data necessary to reproduce the analyses presented here are publicly accessible."

If data are or will be made publicly available, also provide information as to how to access the data. For example: "Data are available from the first author upon reasonable request." Or: "Data are available at the following URL: http://www.example.org"

14) Second, please confirm that one of the following is indicated in the Acknowledgements regarding **analytic code**:

☑ "The analytic code necessary to reproduce the analyses presented in this paper is not publicly accessible."
OR
☐ "The analytic code necessary to reproduce the analyses presented in this paper is publicly accessible."

If code is or will be made publicly available, also provide information as to how to access the code. For example: "Code is available from the first author." Or: "Code is available at the following URL: http://www.example.org"

15) Third, please confirm that one of the following is indicated in Acknowledgements regarding **materials:**

☐ "The materials necessary to attempt to replicate the findings presented here are not publicly accessible."
OR
☑ "The materials necessary to attempt to replicate the findings presented here are publicly accessible"

If materials are or will be made publicly available, also provide information as to how to access the materials. For example: "Materials are available from the first author." Or: "Materials are available at the following URL: http://www.example.org"

16) Fourth and finally, please confirm that that one of the following is indicated in Acknowledgements regarding **preregistration**:

☑ "The analyses presented here were not preregistered."
OR
☐ "The analyses presented here were preregistered."

If analyses were preregistered, also provide information as to how to access the preregistration. For example: "The preregistration is available at the following URL: http://www.example.org"

It is acceptable to combine the steps above for brevity. For example: "The data and code necessary to reproduce the analyses presented here are publicly accessible, as are the materials necessary to attempt to replicate the findings. Analyses were also pre-registered. Data, code, materials, and the preregistration for this research are available at the following URL: http://www.example.org."

MANUSCRIPT TEXT/APA STYLE

☑ 17) ***Italics*****, not underlines**, used throughout text, but only per APA style

☑ 18) **All uses of slash (/) in abstract and body text are removed or edited per APA style** (i.e.,"his/her," "race/ethnicity"; does not apply to references, tables, or figures; see APA Style Manual 7th Ed.)

☑ 19) **Statistics appear in APA style** (e.g., italics, spacing); use either exact *p* levels **or** standard levels only (i.e., .05, .01, .001)

☑ 20) **"Relationship" and "relation"** are used correctly. "Relationship" is used to describe a social bond, while "relation" is used to describe non-animate associations, including those between variables.

PRODUCTION MATERIALS

☑ 21) Submitted a *300–500-word public summary form*.

☑ 22) Submitted *Conflict of Interest forms* signed/completed by all authors.

☑ 23) Submitted a signed/completed *Publications Checklist*.

☑ 24) Submitted *high-resolution, labeled Figures* as PDF, JPEG, TIFF, or EPS files & labelled.

☑ *25) Figures removed from the final file and noted in-text* where each should appear for publication. (Ex. Figure 1 goes here).

Once your paper has been sent to our publisher for production (typically within a few weeks of our office receiving final publication materials) the corresponding author will receive an email from Wiley's Author Services system asking them to log in and complete the appropriate license form.

Please note that publication of your paper is strictly contingent upon completing this form.

PLEASE INITIAL TO CONFIRM THAT:

MPA Research was conducted in accordance with *SRCD ethical standards*.

MPA Any *prior versions of this accepted paper deposited in an archive* or made available for distribution have been designated as 'draft' or 'working paper.'

MPA Authors observed *SRCD methodological recommendations* and adhered to them as fully as possible in the paper. See SRCD webpages for details: https://bit.ly/3ia1siJ

MPA *Conflicts of interest* by any of the authors are explained in the manuscript author notes. The absence of a statement means the authors certify that there are no such conflicts of interest & the authors acknowledge that the editors or publisher may insert a statement to that effect.

MPA Authors have reviewed materials through *StatCheck* (http://statcheck.io), no inconsistent p-values were detected, and authors confirmed as much in their cover letter to the editor.

MPa All authors have seen and *approved the final submission* including information on conflicts of interest.

Your signature below affirms your manuscript is in full compliance with all SRCD Policies described in this checklist.

Corresponding author signature: 28/01/25

Signing Date:

Electronic/Digital Signatures accepted
